# Supplementary figures and images for: Treatment Combining CD200 Immune Checkpoint Inhibitor and Tumor-Lysate Vaccination after Surgery for Pet Dogs with High-Grade Glioma
Source: Cancers (Basel). 2019 Jan 24;11(2):137. doi: 10.3390/cancers11020137 (PMC6406711; doi:10.3390/cancers11020137)

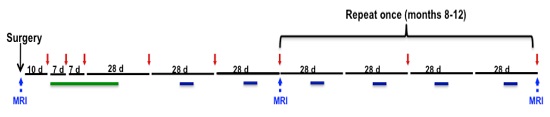

Supplement: Supplementary file 1 [file cancers-11-00137-s001.jpg]
